# Nuclear medicine imaging modalities to detect incidentalomas and their impact on patient management: a systematic review

**DOI:** 10.1007/s00432-024-05891-3

**Published:** 2024-07-25

**Authors:** Roberta Costanzo, Gianluca Scalia, Lidia Strigari, Massimiliano Ippolito, Federica Paolini, Lara Brunasso, Andrea Sciortino, Domenico Gerardo Iacopino, Rosario Maugeri, Gianluca Ferini, Anna Viola, Valentina Zagardo, Sebastiano Cosentino, Giuseppe E. Umana

**Affiliations:** 1 Department of Biomedicine Neurosciences and Advanced Diagnostics, Neurosurgical Clinic, AOUP “Paolo Giaccone”, School of Medicine, University of Palermo, Palermo, Italy; 2 Neurosurgery Unit, Department of Head and Neck Surgery, Garibaldi Hospital, Catania, Italy; 3 Department of Medical Physics, IRCCS Azienda Ospedaliero-Universitaria Di Bologna, Bologna, Italy; 4 Department of Advanced Technologies, Nuclear Medicine and PET, Cannizzaro Hospital, Catania, Italy; 5 Radiation Oncology Unit, REM Radioterapia Srl, Viagrande, Italy; 6 Department of Neurosurgery, Trauma and Gamma-Knife Center, Cannizzaro Hospital, Catania, Italy; 7 Department of Medicine and Surgery, Kore University of Enna, Enna, Italy

**Keywords:** Nuclear imaging, Incidentalomas, PET/CT, Therapeutic strategy, Oncology, Follow-up

## Abstract

**Purpose:**

This systematic review aims to investigate the role of nuclear imaging techniques in detecting incidentalomas and their impact on patient management.

**Methods:**

Following PRISMA guidelines, a comprehensive literature search was conducted from February to May 2022. Studies in English involving patients undergoing nuclear medicine studies with incidental tumor findings were included. Data on imaging modalities, incidentaloma characteristics, management changes, and follow-up were extracted and analyzed.

**Results:**

Ninety-two studies involving 64.884 patients were included. Incidentalomas were detected in 611 cases (0.9%), with thyroid being the most common site. PET/CT with FDG and choline tracers showed the highest incidentaloma detection rates. Detection of incidentalomas led to a change in therapeutic strategy in 59% of cases. Various radiotracers demonstrated high sensitivity for incidentaloma detection, particularly in neuroendocrine tumors and prostate cancer.

**Conclusion:**

Nuclear imaging techniques play a crucial role in detecting incidentalomas, leading to significant changes in patient management. The high sensitivity of these modalities highlights their potential in routine oncology follow-up protocols. Future directions may include enhancing spatial resolution and promoting theranostic approaches for improved patient care.

**Supplementary Information:**

The online version contains supplementary material available at 10.1007/s00432-024-05891-3.

## Introduction

In the twenty-first century, cancer is still considered the second most frequent cause of death after cardiovascular diseases. Through the years, screening methods were increasingly implemented to decrease the mortality rate and to hasten therapeutic strategies. Despite the wide use of CT and MRI imaging, nuclear medicine imaging, indeed, has gained more importance, playing a pivotal role in the diagnostic process and treatment, especially thanks to the use of different radiotracers that can easily detect different malignancies. (Panareo et al. 2021) Indeed, the term “incidentaloma” was introduced to describe the detection of a lesion by chance (either malignant or benignant) while performing an exam for another clinical indication, even leading to the coining of the term “positron emission tomography (PET)‑associated incidental neoplasm” (PAIN) by Katz and Shaha. (Katz and Shaha 2008).

Nuclear medicine imaging techniques, using various radiotracers for different indications, can detect many incidental findings located in different parts of the body. The length of axial field of view (FOV), indeed, may influence scanner sensitivity, requiring a longer FOV to detect images in lesser time and to cover a wider body surface with a lower dose. Thanks to these characteristics, many incidentaloma might be easily detected using these techniques. (Alberts et al. 2023) The most common are in the thyroid, in the gastrointestinal tract, lungs and, in extremely rare cases, in breast. Clearly, an incidental finding can completely change the therapeutic strategies and the approach to the patient and its condition. (Panareo et al. 2021).

Hence, the aim of this systematic review is to investigate and discuss the role of nuclear imaging techniques, their relationship with incidentaloma detection, the advances, and the limitations currently related, and the consequent impact on patient management.

## Materials and methods

### Search strategy

**Fig. 1:**
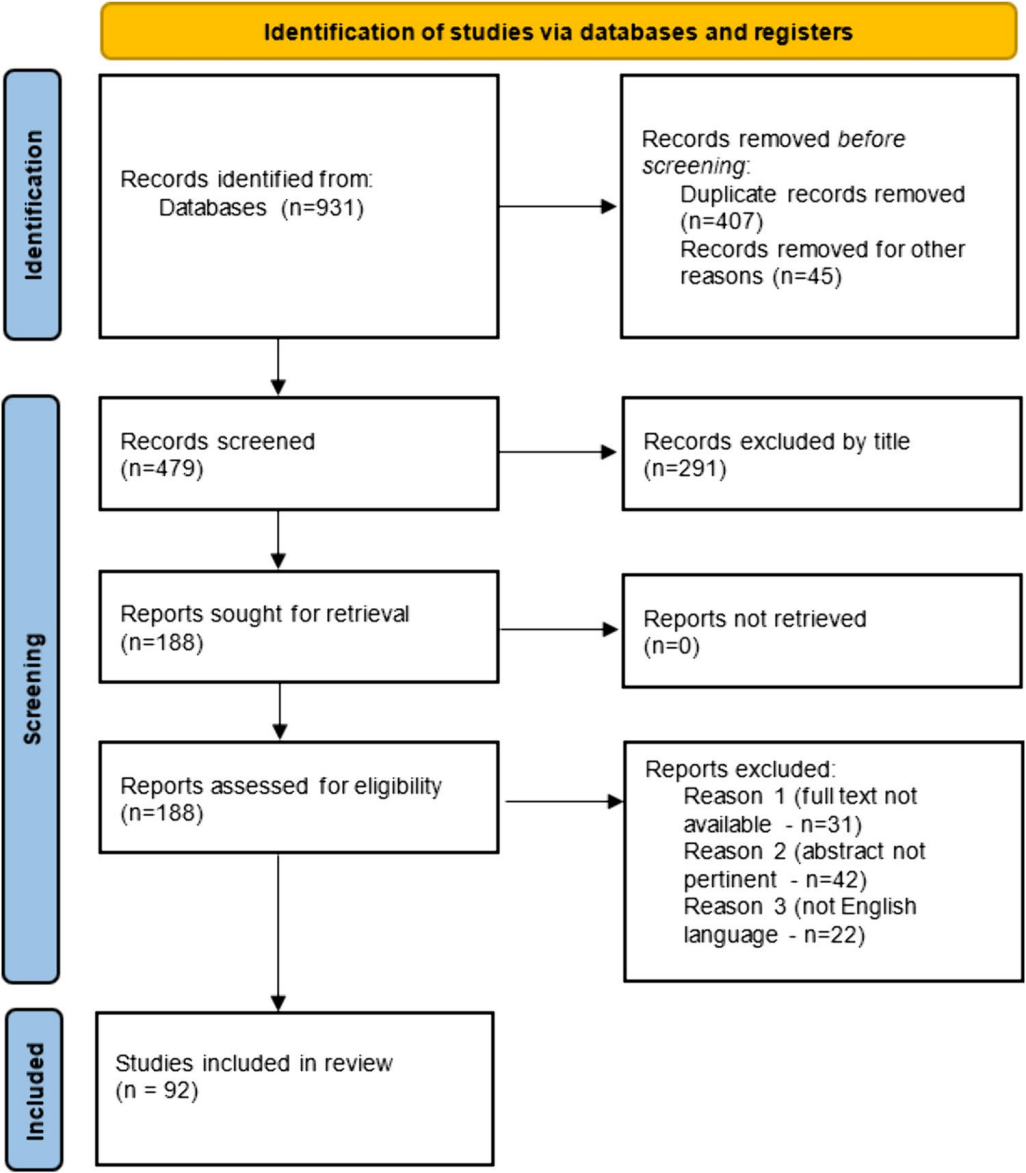
PRISMA diagram depicting the selected studies for this systematic review

An accurate search of the current literature was conducted following the PRISMA (Preferred Reporting Items for Systematic Reviews and Meta-Analyses) **(**Fig. 1**)** statement, with no limits in terms of publication date.

The following Mesh (Medical Subject Headings) terms were used:

“PET FDG AND incidentalomas”, “Positron emission tomography FDG AND incidentalomas”, Whole body PET FDG AND incidentalomas”, “Whole body positron emission tomography FDG AND incidentalomas”, “PET FDG brain AND incidentalomas”, “brain AND PET FDG AND incidentalomas”, “SPECT AND incidentalomas”, “whole body AND scintigraphy AND radioiodine AND incidentalomas”, “Gallium scintigraphy AND incidentalomas”, “Scintigraphy AND Gallium AND incidentalomas”, “Scintigraphy AND Gallium AND incidental tumor”, “Octreoscan AND incidentalomas”, “Octreoscan AND incidental tumor”, “Octreoscan AND incidental lesion”, “111 In-pentetreotide Scintigraphy AND incidentalomas”, “Scintigraphy AND Octreotide AND incidentalomas”, “met PET AND incidentaloma”, “c-met AND incidental”, “c methionine AND incidental”, “methionine PET AND incidentaloma”, “methionine PET AND incidental”, “18 f choline PET AND incidentaloma”, “18 f-choline PET AND incidental”, “choline PET AND incidentaloma”, “choline PET AND incidental”, “choline PET AND incidental lesion”, “choline PET AND incidental tumor”, “choline PET AND incidental uptake”, “68Ga-PSMA PET/CT AND incidentalomas”.

The role of the current review was to analyze different nuclear medicine imaging techniques used to detect incidental lesions, and to evaluate their impact on patient management.

### Eligibility criteria

The following inclusion criteria were applied: studies in the English language, studies involving patients undergoing nuclear medicine studies with incidental findings of primary tumors, age > 18, and presence of clinical data. We deemed eligible for inclusion in this review case reports, cohort prospective and retrospective studies, and clinical trials.

Articles were excluded if they did not deal with clinical management of patients, patients < 18 years old, no detection of incidental tumors, and no English language.

### Selection process

The search was performed from February 2022 to May 2022. The initial search was conducted by four independent authors (FP, RC, LB and AS) using the previously reported keywords. Records were summarized into a single Excel file. All the duplicates were removed. Each author independently screened the titles, abstracts, and full manuscripts using the previously reported selective criteria. Then, the results were analyzed and combined. Another author (GS) also performed a further manual search to identify additional studies of the reference sections of the selected papers.

### Data items

Data extracted from each study were (1) authors, (2) year of publication, (3) number of patients included, (4) sex, (5) age, (6) nuclear medicine imaging modality, (7) study design, (8) reason to perform nuclear medicine imaging modality, (9) number of incidentaloma(s), (10) site(s) of incidentaloma(s), (11) primitive/secondary lesions, (12) histology of incidentaloma(s), (13) timing from surgery, (14) management of incidentaloma(s), (15) change in therapeutic strategy (whenever reported), (16) median follow-up, (17) Standardized Uptake Value (SUV).

## Results

### Study selection

The initial search yielded 931 results. After duplicate removal (452) and screening by title, 291 articles were excluded because they did not meet the inclusion criteria. After a detailed examination of these 188 studies, 95 papers were excluded because the full text wasn’t available (31), the abstract was not pertinent (42), and they were not in English language (22). Hence, a total of 92 studies were included in this literature review. (Fig. 1) (Panareo et al. 2021; Papadakis et al. 2018; Tiktinsky et al. 2011; Gamberini et al. 2011; Cava et al. 2003; Albano et al. 2019, 2017, 2022; Takesh and Adams 2013; Ciappuccini et al. 2018, 2019; Leiris et al. 2018; Florimonte et al. 2016; Cuppari and Evangelista 2018; Tuscano et al. 2014; Hugentobler et al. 2017; Malamitsi et al. 2017; Bagni et al. 2015; Burgers et al. 2018; Treglia et al. 2013, 2015, 2014; Maffione et al. 2016; Cimitan et al. 2013; Vadrucci et al. 2016; Lalire et al. 2016; Calabria et al. 2013; Eccles et al. 2013; Aziz et al. 2015; Imperiale et al. 2013; Paudel et al. 2019; Goineau et al. 2015; Paone et al. 2013; Agrawal et al. 2015; Parekh et al. 2017; Jozanovic and Huic 2022; Freesmeyer et al. 2014; Cegla et al. 2022; Mangili et al. 2019; Sollini et al. 2016; Dondi et al. 2020; Bertagna et al. 2014a, 2013, 2014b; Mapelli et al. 2012; Ouattara et al. 2017; Garzon et al. 2014; Fallanca et al. 2011; Ambrosini et al. 2006; Sasikumar et al. 2017; Demirkol et al. 2017; Leondi Valotassiou et al. 2005; Mannelli et al. 2017; Villa et al. 2003; Punch et al. 2006; Hirano et al. 1993; Al-Hakami et al. 2011; Wan et al. 2019; Zhai et al. 2010; Targe and Basu 2015; Makis and Ciarallo 2017; Kang et al. 2003; Marko et al. 2020; Mihatsch et al. 2021; Makis et al. 2015; Chan et al. 2022; Cohen et al. 2009; Baldane et al. 2015; Seo et al. 2015; Young et al. 2017; Cheen Hoe et al. 2014; Bosch-Barrera et al. 2009; Mullangi et al. 2021; Liu et al. 2020; Inanir et al. 2019; Alçin et al. 2019; Plouznikoff et al. 2019; Pfob et al. 2019; Militano et al. 2019; Vaz et al. 2018; Malik et al. 2018; Farolfi et al. 2018; Singh et al. 2018; Krishnaraju et al. 2018; Dias and Bouchelouche 2018; Patel et al. 2017; Lawhn-Heath et al. 2017; Bilgin et al. 2016; Chan and Hsiao 2016; Lasocki et al. 2016; Maffei et al. 2012; Cleary et al. 2016).

### Study characteristics

A total of 92 studies were involved in statistical analysis. Regarding the study design, most were case reports (72/92). The remaining studies were 4 case series, 14 retrospective studies, 2 prospective studies and 1 population study. A total of 64,884 patients were involved. 611 incidentalomas were detected (0.9%). (Suppl. 1).

Patients’ demographics and main characteristics are summarized here. (Suppl. 2).

### Statistical analysis

All the selected records were used to structure the database to evaluate the role of nuclear medicine imaging modalities in detecting incidental lesions and their impact on patients’ management. Nuclear medicine imaging modalities were different:

–99mTc-Sestamibi, 2 studies
- 99mTc-colloid, 1 study
- I-6-iodomethyl-norcholesterol (131I-NP-59 [NORCHOL-131]; CIS SPECT, 1 study
- 18F choline and 11C choline, 40 studies
- 18F-Choline & 18F-FDG, 1 study
- 68 Ga PSMA, 17 studies
- 111 In-pentetreotide Scintigraphy, 3 studies
- 67 Ga Scintigraphy, 2 studies
- 18F-FDG, 19 studies
- 68Ga-DOTATATE, 5 studies
- 68Ga-DOTATOC, 1 study

The clinical series included 64,884 patients. The total number of incidentalomas detected was 611 (0.9%). 3517 (5.4%) patients were men and 2645 (4%) were women. (Table 1) Sex was not reported in 3 studies (58,722 patients, 91%). The M: F ratio was 1.3:1. The mean age was 64.1 years.

**Table 1:** Number of patients and incidentalomas by nuclear medicine methodics

Nuclear imaging modalities	Sum of N of patients	Sum of N incidentaloma(s)	Sum of N of studies
111In-Pentetreotide	3 (0,005%)	5 (0.81%)	3 (3.26%)
131I-NP-59	47 (0.07%)	47 (7.7%)	1 (1.08%)
18F-Choline and 11C-Choline	1757 (2.7%)	84 (13.74%)	40 (43.47%)
18F-Choline & 18F-FDG	1 (0.001%)	1 (0.16%)	1 (1.08%)
18F-FDG	62,882 (97%)	438 (71.7%)	19 (20.6%)
67 Ga Scintigraphy	2 (0.003%)	2 (0.3%)	2 (2.2%)
68 Ga PSMA	17 (0.026%)	17 (2.8%)	17 (18.5%)
68 Ga-DOTATATE	26 (0.04%)	6 (1%)	5 (5.4%)
68 Ga-DOTATOC	38 (0.06%)	1(0.16%)	1(1.08%)
99mTc-Sestamibi	110 (0.17%)	9 (1.5%)	2 (2.2%)
99mTc-colloid	1 (0.001%)	1(0.16%)	1(1.08%)
Grand total	64,884	611	92

*18F-FDG* [18F]Fluorodeoxyglucose, *PSMA* prostate-specific membrane antigen, *131I-NP59* Iodine-131 6β-Methyl-Iodo-19 Norcholesterol, *99mTc-Sestamibi* Technetium 99m-methoxy isobutyl isonitrile

Of the incidentalomas detected, 611 were primitive lesions and the most common site was the thyroid.

The updated analysis of 611 incidentalomas detected across various nuclear medicine imaging modalities revealed diverse findings. Sestamibi SPECT/TC identified 1 incidentaloma (0.16%) in the pulmonary area, while Sestamibi TC detected 8 incidentalomas (1.31%) in the pituitary gland, liver, and submandibular gland. There was a single incidentaloma (0.16%) of myelolipoma found using 99-Tc SPECT/TC, and 47 incidentalomas (7.69%) in the adrenal gland by I-131 iodomethyl-norcholesterol (131I-NP-59 [NORCHOL-131]; CIS SPECT). 18F choline PET/CT detected 9 incidentalomas (1.47%) in the thyroid. Furthermore, 18F choline PET identified 47 incidentalomas (7.69%) in various sites, including thymic carcinoma, papillary thyroid carcinoma, large B-cell lymphoma, ischiatic solitary plasmocytoma, salivary gland carcinoma, high-grade (G3) mucinous colon carcinoma, breast cancer, acoustic neurinoma, colorectal cancer, pituitary macroadenoma, Leydig cell tumor in the testis, left tonsil squamous cell carcinoma, right breast carcinoma, colon carcinoma, pulmonary NET, adrenocortical adenoma, brain plasma cell neoplasm, lymph node Hodgkin lymphoma, thyroid Hürthle cell adenoma, neurofibroma in S2 to S4 sacral nerve roots, bladder carcinoma, parathyroid adenoma, schwannoma near the Achilles tendon, laryngeal squamous cell carcinoma, and lung melanoma.

11C choline PET/CT found 7 incidentalomas (1.15%) including esophageal squamous cell carcinoma, frontal lobe meningioma, parathyroid adenoma, papillary thyroid carcinoma, inguinal lymph node large B-cell lymphoma, thymoma, and ilium plasmocytoma. 68Ga PSMA-PET/CT detected 31 incidentalomas (5.07%) across various sites, including the stomach, medullary thyroid cancer, primary pancreatic cancer, vestibular schwannoma, liposarcoma, GIST, thymic carcinoma, pheochromocytoma, thymoma, lumbar schwannoma, left orbitofrontal region, and right superior cerebellar lesion.

In-111 pentetreotide Scintigraphy identified 3 incidentalomas (0.49%) of meningioma, medullary thyroid cancer, and thyroid follicular cancer, while 67 Gallium Scintigraphy found 2 incidentalomas (0.33%) of invasive ductal carcinoma and meningioma.

Whole-body FDG-PET/CT was the most prolific, detecting 115 incidentalomas (18.82%) across a wide range of sites including the thyroid, parotid, cervical lymph node, lungs, head and neck region, colon, pelvis, adrenal glands, bone, esophagus, inguinal area, kidney, liver, mediastinum, prostate gland, rectum, and stomach.

68Ga-DOTATATE identified 2 incidentalomas (0.33%) of tonsillar lesion and pelvic, while 68Ga-DOTATATE PET/CT detected 19 incidentalomas (3.11%), including pituitary uptake, retroperitoneal liposarcoma, vestibular schwannoma, left internal obturator muscle, parathyroid gland, liposarcoma, GIST, thymic carcinoma, pheochromocytoma, thymoma, lumbar schwannoma.

A Gallium-68 DOTA-octreotate CT/PET found 1 incidentaloma (0.16%) in the left cerebellar hemisphere near the vermis, and 68Ga-DOTATOC PET/CT identified 1 incidentaloma (0.16%) of meningioma. Additionally, whole-body FDG-PET/CT reported 1 incidentaloma (0.16%) of pituitary gland.

In 10 (11%) studies, nuclear medicine imaging was performed before surgery instead of 20 after surgery. In 10 (11%) studies, patients did not perform surgery, and 55 (60%) studies did not report the data. **(**Table 2**).**

**Table 2:** Summarizes the indication for nuclear medicine imaging and the number of incidentalomas detected

Indication for imaging	Sum of N incidentaloma(s)
Clinical suspicion	18 (3%)
Clinical suspicion/ Symptomatic patient	51(8.3%)
Cancer screening or Routine follow-up	76 (12.4%)
Routine follow-up	390 (64%)
Symptomatic	8 (1.3%)
Not reported	37 (6%)
Others: drug safety clinical trials	31 (5%)
Grand total	611

After finding these lesions, 53 studies (58%) including 358 incidentalomas (59%) reported a change in therapeutic strategy, 4 studies (4.3%) including 74 incidentalomas (12%) did not change strategy, and, finally, 34 studies (37%) including 179 incidentalomas (29%) did not report these details. **(**Table 3**)** Incidentalomas were treated with chemotherapy, radiotherapy, a combination of radio and chemotherapy, surgery, Gamma Knife or only with follow-up. **(**Table 4**)** The median follow-up of the patients was 0.5 years.

**Table 3:** Change in therapeutic strategy versus the number of incidentaloma(s)

Change in therapeutic strategy?	Sum of N Incidentaloma(s)
Yes	358 (59%)
No	74 (12%)
Not reported	179 (29%)
Grand total	611

**Table 4:** Summarizes interventions in case of changes in therapeutic strategy following incidentaloma detection

If yes, what?	Sum of N incidentaloma(s) %
Biopsy	1(0.28)
Chemotherapy	1(0.28)
Follow-up	1(0.28)
Micro biopsy	1(0.28)
Radiotherapy	3 (0.84)
Radiotherapy and chemotherapy	1(0.28)
Surgery	169 (47)
Surgery & hormonal therapy	1(0.28)
Not reported	180 (50)
Grand total	358

The mean SUV at baseline of the incidentalomas in the different studies was 6.6, ranging from 2.4 to 41.4, in the overall population. We also analyzed the SUV measurements across various nuclear medicine imaging modalities. For 18F choline PET/CT, SUV values ranged from 3.1 to 12, with notable averages of 9.6 and 4.5 for malignant lesions. In contrast, benign lesions showed lower SUV values. Similarly, 18F choline PET showed SUV values ranging from 2.4 to 11 across different studies. 11C choline PET reported an SUV of 6.0. 68 Ga PSMA-PET/CT results varied widely, with SUV values between 3.1 and 24.5. Whole-body FDG-PET/CT showed significant variations, with SUV values reaching as high as 41.4. 68 Ga-DOTATATE PET/CT and 68 Ga-DOTATOC also demonstrated varied SUV values, highlighting the diverse metabolic activity detected across different imaging modalities. These SUV values vary significantly across different imaging techniques and types of lesions, underscoring the importance of modality-specific interpretation of SUV measurements in clinical practice.

## Discussion

PET in oncological diagnosis is gaining a pivotal role, transforming PET, PET/CT, and scintigraphy into essential imaging tools for detecting and staging malignant lesions. Various radiotracers, linked to different pathophysiological pathways, can identify changes in cellular metabolism, thereby detecting neoplastic tissue. These radiopharmaceutical isotopes are highly specific and absorbed by neoplastic hypermetabolic cells. PET or PET/CT and scintigraphy are primarily used for diagnosis and tumor staging, and an incidental finding, such as a second malignant lesion, can significantly alter medical strategies and patient management (Katz and Shaha 2008; Alberts et al. 2023).

This is an interesting issue we would like to address in this systematic review. This review revealed which nuclear imaging techniques can detect the higher rate of incidentalomas and the implication of this casual discovery to determine the best therapeutic strategies for patients’ care.

Literature data show that the most common incidental findings are in the thyroid, gastrointestinal tract, and lungs, with an incidence ranging from 1% to 3%, with a clear prevalence for thyroid incidentalomas. According to our literature review, the most represented imaging technique capable of detecting an incidentaloma is PET/CT with the following radiotracers: 18F-fluoro-2-deoxy-D-glucose (FDG) and 18F-choline. The total number of incidentalomas reported here (611) may appear to be exiguous, accounting for 0.9%, but we believe this data is underestimated and deserves further interpretation.

First, the use of PET/CT is limited due to its costs, device availability, and lack of protocols supporting its mandatory use. Second, the increasing use of modern radiosurgical and stereotactic radiotherapy techniques, such as gamma knife, cyberknife, and proton beam, both with single fraction or hypofractionated regimes, requires routine use of nuclear medicine imaging modalities to detect the clonogenic microscopic infiltration of the tumor that is not visible with standard imaging. This microscopic infiltration affects the feasibility of the treatment, as reported in our previous studies (Albano et al. 2022; Inserra et al. 2022; Barone et al. 2021). Radiosurgical treatments may not be suitable if PET/CT shows extensive bony infiltration, not visible on MRI scans. Similarly, the detection of multiple lesions not documented on MRI changes the SRS plan and dose delivery (Albano et al. 2022). Since the use of SRS treatments is increasing and with this the related nuclear medicine investigation, we can assume that the number of incidentalomas reported may rise in the near future.

In this literature review emerged that the detection of incidentalomas lead to a change in the therapeutic strategy in hundreds of patients, accounting for 59% of incidentaloma. Also, this undoubtful consistent data may be even underestimated, since a large number of papers didn’t report detailed information on the management of 179 new discovered lesions (29%).

Of notice, most of the tracers reported appear to be highly sensitive for incidentalomas detection. Of 47 patients that performed 131I-NP-59 PET scan, all 47 showed incidentalomas. (Cava et al. 2003) The same for all 17 68Ga PSMA-PET/CT that showed 17 incidentalomas patients, and 67Ga Scintigraphy, 99mTc-colloid. (Sasikumar et al. 2017; Ciappuccini et al. 2019; Demirkol et al. 2017; Punch et al. 2006; Hirano et al. 1993) Similarly, all the other tracers demonstrated the capability to detect new lesions and in particular FDG-PET showed a significant rate of incidentalomas detection, especially if considered that this investigation is widely used in oncology for several histotypes. Despite FDG-PET documented a low incidence of new lesions, its diffuse application led to a high number of incidentalomas.

68Ga-DOTATATE and DOTATOC showed a relatively lower number of incidentalomas, (Chan and Hsiao 2016; Cleary et al. 2016; Albano et al. 2022) but it should be considered that these investigations are used only in selected cases, mainly in the assessment of meningiomas and neuroendocrine tumors and in particular for Gamma knife (GK) planning. Thus, the reported 4% of new lesions is not negligible especially considering that not all GK center perform 68Ga-DOTATATE or DOTATOC scans due to lack of clear protocols and of tracer availability.

Another question that deserves to be mentioned are the new application of tracers for histology other than the usual, like 68Ga PSMA-PET/CT that is commonly used in prostate cancer but that has been reported to be beneficial in radiotherapy contouring of patients affected by gliomas due its highly sensitivity for the detection of glioblastoma infiltration (Şahin et al. 2022). 68Ga PSMA-PET/CT is able to highlight glioma infiltration not visible with standard imaging, similarly to 68Ga-DOTATATE or DOTATOC that can detect the clonogenic infiltration, and thus allowing for a more precise definition of tumoral edges crucial during radiotherapy contouring. (Umana et al. 2022)

The analysis of SUV values across various nuclear medicine imaging modalities reveals significant variability, underscoring the importance of interpreting these values within the context of each specific modality. For instance, 18F choline PET/CT showed SUV values ranging from 3.1 to 12, with higher values typically indicating malignant lesions. In contrast, benign lesions had lower SUV values. Similarly, 68Ga PSMA-PET/CT displayed a wide range of SUVs from 3.1 to 24.5, demonstrating its sensitivity in detecting various incidentalomas, including glioma infiltrations. Whole-body FDG-PET/CT exhibited even greater variability, with SUVs reaching up to 41.4, reflecting the hypermetabolic activity common in many malignancies. These differences highlight the necessity for modality-specific interpretation of SUV measurements, as accurate assessment is crucial for identifying incidentalomas and making informed changes in therapeutic strategies. This tailored approach ensures that incidental findings are effectively characterized, leading to better patient management and outcomes.

### History of PET

Positron emission tomography is an imaging technique born in the late 1970s, thanks to the discovery by Otto Warburg that malignant cells have a higher glycolytic rate. Indeed, FDG is an analogue of glucose that is carried across the cell membrane and once phosphorylated, is metabolically trapped. Due to this similarity, ^18^FDG can be used as an excellent radiotracer for PET. All the preliminary PET studies have started studying brain disorders and the existence of specific neurotransmitters has introduced various biomarkers, highly specific for different pathologies.

Since the 1980s, after the discovery that many tumoral lesions are associated to an increased uptake of glucose, the application of PET, using ^18^FDG, has gained a progressive central role in the oncological field, first detecting and staging lung tumors, subsequently including thyroid, breast, lymphoma, colorectal, esophageal cancer, and melanoma.

To characterize glucose metabolism, a standardized uptake value (SUV) was created, identifying into the lesion a region of interest (ROI) dividing the value by the injected activity and then dividing it by patients’ body weight.

Through the years, the resolution of PET images has rapidly improved, until the combination with CT scanners and MRI guarantees an outstanding quality of images (Rohren et al. 2004).

The most used radiotracers that have detected the highest number of incidentalomas are the following:

- 18F‑fluoro‑2‑deoxy-dglucose (FDG) is the most used radiotracer but not the only one, developing several radiopharmaceuticals highly specific for different tissues and part of the body. It is still most used in detection of breast lesions, pituitary lesions.
- Several case series have reported the role of FDG in the identification of pituitary lesions, mostly macroadenomas (Bertagna et al. 2019). Nevertheless, in case of ACTH or GH microadenoma, aminoacidic PET tracer has shown an undeniable superiority.
- Thyroid incidentalomas detection using this methodic was largely investigated, especially by Hsieh et al. and by King et al. (the last one reported the lowest number of incidentaloma using this radiotracer, probably due to a lower risk of thyroid disease related to the geographical area examined) (King et al. 2007).
- In the study of Kang et al. (2009), instead, were reported 1151 incidentalomas over 12,840 patients. According to all these studies, indeed, almost one third of patients were asymptomatic and have received a completely incidental diagnosis that may have hasten a slower investigation of thyroid malignancies (Bertagna et al. 2019).
- Choline is a lipid, collected into cells membranes and a useful marker in spectroscopy to diagnose brain, prostate, lung, breast, and esophageal tumors. First, ^11^C choline was the most used; recently, ^*18*^* fluorocholine* (FCH), has shown similar characteristics as radiotracer and better results due to a longer half-life. Its uptake was observed both in benign pathology, as parathyroid adenoma, frequently found incidentally during prostate cancer follow-up and, also, in slow growth lesions that can be missed with FDG. Interestingly, according to Calabria et al., ^18^F-choline can detect inflammatory (80/1000 patients), hypermetabolic (7/1000) lesions due to a presumed tracer accumulation into macrophages. (Broos et al. 2022; Bertagna et al. 2019; Hodolic et al. 2014) Hodolic et al., has reported 8 cases, including 2 meningiomas and 3 pituitary adenomas, detected in patients with prostate cancer during routinary follow-up. This tracer can be also useful in low grade tumor and meningiomas diagnosis, due to a lower uptake in normal brain parenchyma. Especially in case of prostate cancer, an incidental diagnosis of brain meningioma can change radically the therapeutic strategy, due to the high risk of tumor development using anti-androgen therapy(Hodolic et al. 2014). This observation has clearly proven the pivotal role of “PAIN” findings.
  - ^11^C methionine and ^11^C acetate are also valuable amino acids used as positron-emitting isotopes for the evaluation of prostate cancer and brain tumors (Rohren et al. 2004).
  - Gallium-68 as DOTA-Phe1-Tyr3-Octreotide (68 Ga-DOTATOC, DOTA-TATE, DOTA-NOC PET/TC) has been mostly used for the evaluation of neuroendocrine, renal cells, prostate tumors, or malignant lymphoma, medulloblastoma, neuroblastoma and sarcoma that have a high expression of somatostatin receptor. 67 Gallium, instead, is mostly used for the diagnosis of lymphoma or to identify spinal infection.
  - 68Ga-DOTATOC has shown a great efficacy detecting SSTR2-positive incidental benign lesions, such as meningiomas, or metastatic lesions, not recognizable using standard imaging modalities. This imaging technique, indeed, may guarantee an early diagnosis for planning Cyber Knife and Gamma Knife radiosurgery. Moreover, it can be useful to detect multiple lesions in cases of neurofibromatosis, harvesting patients’ management (Umana et al. 2022).
  - PSMA (prostate-specific membrane specific antigen) is expressed mainly in prostate cancer. However, its expression can be identified in other malignancies such as colon, gastric, thyroid, and renal cancer, breast and in the neovasculature of benign lesions (Dias and Bouchelouche 2018; Patel et al. 2017; Lawhn-Heath et al. 2017; Bilgin et al. 2016).

### Scintigraphy

Scintigraphy is a nuclear medicine diagnostic test, where radioisotopes attached to drugs that travel to a specific organ or tissue are internalized and the emitted gamma radiation is captured to form two-dimensional images. This methodic with the somatostatin receptor agent In-111 pentetreotide is most useful to detect patients with neuroendocrine tumors (APUDoma), but the tumor-targeting potential of this radiopharmaceutical in other types has been also described in literature. (Leondi Valotassiou et al. 2005; Mannelli et al. 2017; Villa et al. 2003) Villa et al. described a peculiar case of a 38-year-old female patient with lung carcinoid that underwent scintigraphy with In-111 pentetreotide, positive in the known lung tumor and incidentally revealed uptake in a thyroid nodule, with a final diagnosis of follicular cancer. (Villa et al. 2003) Leondi et al. also reported an uptake of In-111 in the right cerebral hemisphere, consisting of meningiomatosis. (Leondi Valotassiou et al. 2005).

Furthermore, another nuclear medicine methodic, the whole-body gallium-67 scintigraphy, is routinely performed to evaluate localization of source of fever in cases of fever of unknown origin (FUO), sarcoidosis, tuberculosis, retroperitoneal fibrosis, spinal osteomyelitis, and other conditions. (Punch et al. 2006; Hirano et al. 1993) Punch et al. incidentally discovered an abnormal focus of tracer uptake in the retroareolar region of the right breast during a whole-body gallium-67 scintigraphy to detect an occult infection. Subsequent biopsy of the right breast lesion revealed an invasive ductal carcinoma. (Punch et al. 2006).

### Future direction and limitation

In the wide and potential application of PET, there are still some technical limitations that may be considered. For example, the use of radiations in humans is limited by reluctance and fear of radiation absorption, despite the proven of radiolabeled tracers. This issue has strongly limited the use of this imaging technique, especially as screening in healthy patients.

In the future, the use of kinetic signatures and the improvement of spatial resolution could lead to detect existing disease expressed as a separate tracer kinetic element, below the spatial resolution of the scanner. This improvement could be applied both in infection and in oncology (Jeong et al. 2010).

Moreover, the increasing use of PET in the oncological field should require shorter time per patient, restricting the volume of patients scans. For this reason, the goal should be obtaining more sensitive whole-body PET scanners, to process more patients in less time.

Another goal that may be achieved in the upcoming future is to significantly promote the role of theranostic (i.e., a combination of therapy and diagnostic) in all fields of PET-based oncology (Jeong et al. 2010; Jones and Townsend 2017).

## Conclusion

Nuclear imaging techniques play a pivotal role in the detection of incidentalomas, offering crucial insights that often lead to significant changes in patient management strategies. The findings from this systematic review underscore the high sensitivity of modalities such as PET/CT, particularly with FDG and choline tracers, in uncovering unexpected lesions across various malignancies. With thyroid, gastrointestinal, and pulmonary sites being commonly affected, the implications of incidentaloma detection extend beyond mere diagnosis, often prompting immediate adjustments in therapeutic approaches. The integration of nuclear imaging into routine oncological follow-up protocols holds immense promise for enhancing patient care and tumor control. However, future advancements in spatial resolution and the continued exploration of theranostic applications are warranted to further optimize the clinical utility of these techniques. Overall, the growing body of evidence presented here underscores the indispensable role of nuclear imaging in modern oncology, paving the way for more personalized and effective patient management strategies.

### Supplementary Information

Below is the link to the electronic supplementary material.

Supplementary file1 (XLSX 9 KB)

Supplementary file2 (XLSX 120 KB)

## Data Availability

No datasets were generated or analysed during the current study.
